# Ethnoichthyology of the indigenous Truká people, Northeast Brazil

**DOI:** 10.1186/s13002-015-0076-5

**Published:** 2016-01-06

**Authors:** Carlos Alberto Batista Santos, Rômulo Romeu Nóbrea Alves

**Affiliations:** Programa de Pós Graduação em Etnobiologia e Conservação da Natureza, Departamento de Ciências Biológicas, Universidade Federal Rural de Pernambuco, Rua Dom Manoel de Medeiros, s/n, Dois Irmãos, 52171-900 Recife, PE Brasil; Departamento de Tecnologia e Ciências Sociais, Universidade do Estado da Bahia, Avenida Edgard Chastinet, s/n, São Geraldo, 48905-680 Juazeiro, BA Brasil; Departamento de Biologia, Universidade Estadual da Paraíba, Av. das Baraúnas, 351/Campus Universitário, Bodocongó, 58109-753 Campina Grande, Paraíba Brasil

**Keywords:** Ethnobiology, Ethnozoology, Fisheries, São Francisco river, Traditional knowledge

## Abstract

**Background:**

Historically, fishing is an important activity for riverine communities established along the São Francisco River, including indigenous communities. In the present study, we researched fishing activities in two villages of the Truká ethnic group, both located in the State of Pernambuco along the sub-middle section of the São Francisco River, Northeast Brazil. We recorded the richness and uses of the fished species and the ecological knowledge on these species, the fishing techniques employed and the perception of the indigenous people regarding current environmental impacts on the São Francisco River that influence local fishing.

**Method:**

The information was obtained through interviews with 33 Truká fishers (27 men and six women), including 17 interviewees from Central Village (Cabrobó) and 16 from Tapera Village (Orocó).

**Results:**

Using five fishing techniques, the interviewees caught 25 fish species, including 21 native and four exotic species. All species are used as food, and two species are used in traditional Truká medicine. The interviewees revealed that fishing currently has less importance in their subsistence. They indicated that this situation is occurring because of several factors, such as the introduction of exotic species, pollution and urbanization, that have impacted the São Francisco River, resulting in a decline of fishing resources. Nevertheless, we found that the indigenous people who are still fishing have a broad knowledge of the habitat and ecology of the target fishing.

**Conclusion:**

Although fishing is declining in importance among the Truká, we found that the individuals who are still practicing this activity have a broad knowledge about the habitat and ecology of the target species and apply that knowledge to fishing methods. Knowledge about the ecology of the species and the environmental impacts that have affected them can support basic research on local fish populations and research investigating the environmental impacts, resource management and sustainable exploitation of fisheries resources.

## Background

Fishing is one of the oldest activities in human history [1] and has continued to play an essential role in the subsistence, economy and culture of many human communities worldwide [2–8]. In Brazil, home to a large diversity of coastal and continental aquatic ecosystems, fishing activities are employed by indigenous communities and by different traditional communities that were formed during the European colonization, persisting in many regions of the country to the present as an activity of great social and cultural importance [9].

Artisanal fishers are recognized for having developed an elaborate knowledge about the biological resources exploited, which includes aspects of ecology, taxonomy and ethology [10–14]. This knowledge has been examined through ethnozoological studies, which indicate that information from fishers can support academic research on the biology of the exploited species or research directed toward the development of management and sustainability plans for the exploited resources [15–23].

Considering the importance of fishing in Brazil, many ethnoichthyological studies have been conducted in the country, especially in the riverine fishing communities of the Amazon, in the *Caiçara* communities of the south-eastern coast and in the estuarine-marine areas of the northeast [19]. In reservoirs and rivers within the Caatinga (Brazilian savanna) morphoclimatic domain, a few studies have been conducted on the exploitation of fishing resources by artisanal fishing communities. Usually, these studies have focused on the fishers from the banks of the São Francisco River [24–27].

Ethnoichthyological studies involving indigenous populations of the Brazilian semi-arid regions are rare, although many indigenous ethnic groups (i.e., the Tuxá, Pankararu, Pankararé, Kantaruré, Xucuru Kariri, Atikum and Truká) have practiced fishing for many years. Fishing has historical importance, especially for the ethnic groups settled along the São Francisco River, where, naturally, they had access to the river wildlife, which made fishing one of the main sources of protein for these communities [28]. This scenario, however, has undergone profound changes in recent decades with the damming of the São Francisco River in many areas, leading to hydrological alterations and flooding of the territories of these indigenous populations. Thus, fishing, once an activity of paramount importance for these populations, has lost its leading role.

This study is the first ethnoichthyological research conducted with the Truká ethnic group inhabiting the semi-arid region of the State of Pernambuco. This is a descriptive study that sought to 1) assess the richness of the fished species and the fishing techniques employed by these people, 2) investigate whether fishing remains an important activity as a source of protein, and 3) analyse the local knowledge on the fished species that are important in the organization of fishing activities. Moreover, the present study also considered the perception of the indigenous people regarding the alteration in fishing practices resulting from changes made in recent years in the São Francisco River with the construction of local hydroelectric plants.

## Methods

### Study area

**Fig. 1:**
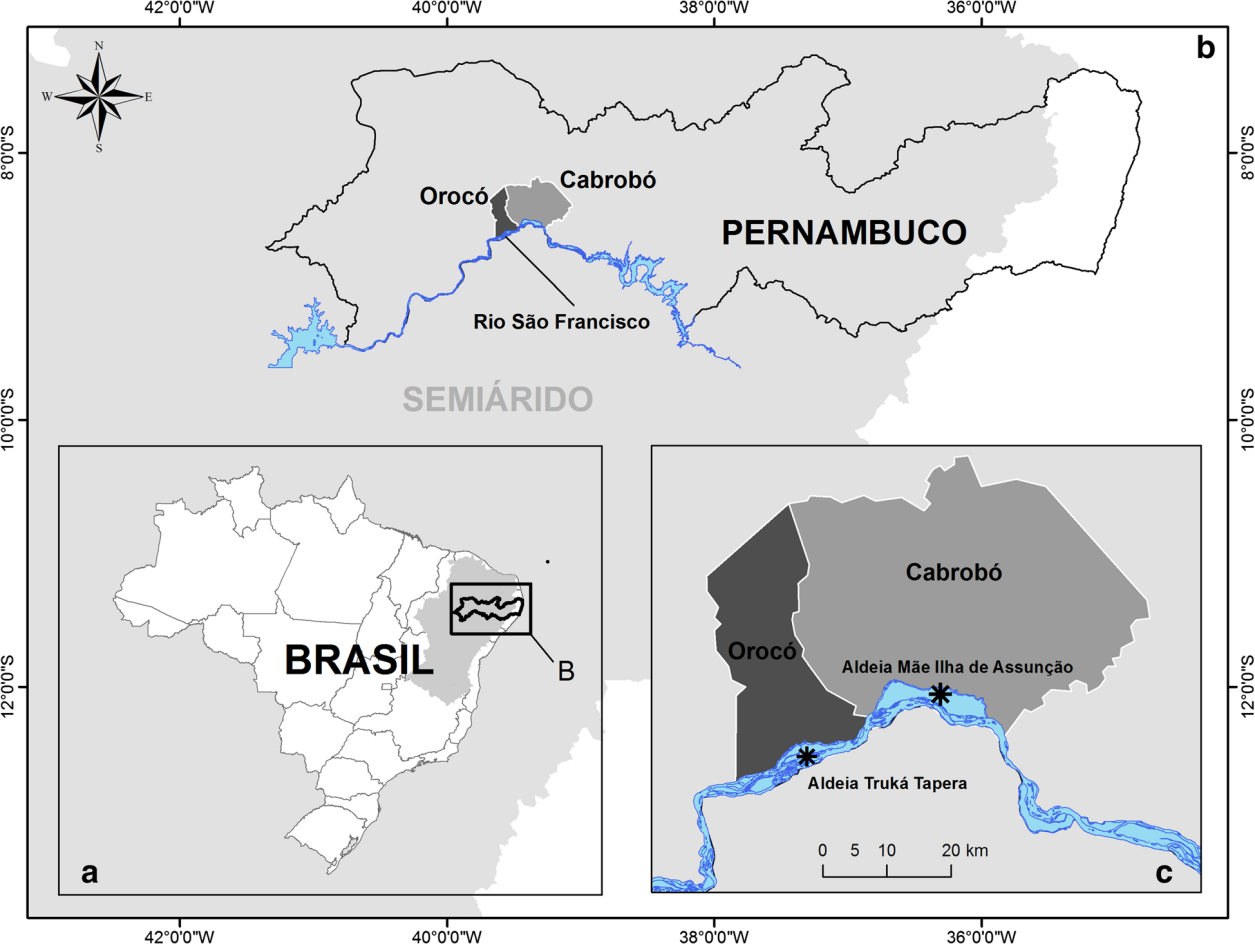
Locations of Truká villages: Central village (Cabrobó) and Truká Tapera Village (Orocó) (**c**), Pernambuco State (**b**), Brazil (**a**)

The study was conducted in two Truká villages located in the north-eastern semi-arid region, along the sub-middle section of the São Francisco River, State of Pernambuco, Brazil (Fig. 1). One of the communities, called Aldeia Mãe (Mother Village), is located in Assunção Island, municipality of Cabrobó (8° 31′ 07.11′′ S x 39° 22′ 20.87′′ W), and the other village is located in Tapera Island, municipality of Orocó (8° 36′ 24.4′′ S x 39° 34′ 54.9′′ W), 39.85 km from Aldeia Mãe. Both villages are located within the Caatinga domain [29, 30], where the main economic activity is agriculture, followed by animal husbandry and handicraft production. Fishing and hunting are elements of the cultural tradition that identify and differentiate the ethnic groups [31].

### Legal and ethical aspects

Considering the ethical aspects, before each interview, the purposes of this present study were explained and permission requested to register the information through the presentation and signing of the Informed Consent Form (ICF) and the authorization of image use form. The authorization to access the traditional knowledge associated to the genetic heritage was granted by the Research Ethic Committee (Legal view N°723.750), of the National Historic and Artistic Heritage Institute (N° 013/2013 legal process n° 01450.010527/2013-30), and the clearance to ingress in Indian territories was granted by the National Indian Foundation, supported the Lower São Francisco Regional Coordination.

### Data collection

The data were collected from July 2013 to February 2014, with an effort of 4 days per month in each village surveyed, totalling 32 days for each village over the course of the study. The information was obtained through interviews with 33 Truká fishers (27 men and six women), including 17 interviewees from Aldeia Mãe (Cabrobó) and 16 from Tapera Village (Orocó).

A non-probabilistic purposive sampling method was applied to select the interviewees [32] using the snowball technique [33]. Indigenous fishermen and fisherwomen living in the villages, who were 18 years of age or older, were interviewed. Information on traditional ecological knowledge and the local use of the ichthyofauna was obtained through semi-structured questionnaires using free interviews and informal conversations [32, 34]. Questionnaires contained questions about the fish species caught, fish uses, capture methods and habitat of the fished species.

Specimens were identified through consultation with experts, through examination of specimens acquired directly from the fishers and through photographs taken during the interviews. All specimens were deposited in the Zoology Museum, Fish Division, Feira de Santana State University (Universidade Estadual de Feira de Santana-UEFS). Questionnaires, photographs, and recorded interviews were deposited in the Opará Indigenous Training and Research Centre (Centro de Formação e Pesquisa Indígena Opará), Bahia State University (Universidade do Estado da Bahia-UNEB).

## Results and discussion

### Species used

The Truká interviewed cited a total of 25 fish species used in the villages surveyed. Higher species richness (*n* = 24) was mentioned in the Cabrobó village compared to the Orocó village (*n* = 15) (Table 1). The most cited species were *Myleus micans* (pacu) (*n* = 30 citations), *Leporinus* cf *piau* (piau) (*n* = 22), *Leporinus obtusidens* (true piau) (*n* = 21), *Prochilodus argenteus* (crumatá) (*n* = 19) and *Metynnis maculatus* (spotted metynnis) (*n* = 18).

**Table 1:** Fish species used by the Truká people from the Cabrobó (Aldeia Mãe) and Orocó villages

Family/Local species name	Scientific species name	Origin	No. of citations (villages)	
			CA	O
Sciaenidae				
Pescada	*Plagioscion squamosissimus* Heckel, 1840	E	10	10
Cichlidae				
Apairi	*Astronotus ocellatus* Agassiz, 1831	E	5	–
Tucunaré	*Cichla ocellaris* Bloch & Schneider, 1801	E	8	9
Serrasalmidae				
Tambaqui	*Colossoma macropomum* Cuvier, 1818	E	10	4
Characidae				
Pirambeba	*Serrasalmus brandtii* Reinhardt, 1874	N	8	5
Piranha	*Pygocentrus piraya* Cuvier, 1819	N	6	5
Pacu	*Metynnis maculatus* Kner, 1860	N	8	10
Pacu-preto	*Myleus micans* Reinhardt, 1874	N	15	15
Dourado	*Salminus* cf*. brasiliensis* Cuvier, 1817	N	8	–
Erythrinidae				
Traíra	*Hoplias malabaricus* Bloch, 1794	N	6	4
Bryconidae				
Matrinchã	*Brycon reinhardtii* Lütken, 1875	N	3	–
Anastomidae				
Piau	*Leporinus* cf *piau* Fowler, 1941	N	9	13
Piau-verdadeiro	*Leporinus obtusidens* Valenciennes, 1836	N	6	15
Prochilodontidae				
Crumatá	*Prochilodus argenteus* Agassiz,1829	N	12	7
Loricariidae				
Cananã	*Hypostomu*s *margaritifer* Regan, 1808	N	5	8
Cascudo	*Pterygoplichthys etentaculatus* Spix & Agassiz, 1829	N	6	–
Xotó	*Hypostomu*s *macrops* Eigenmann & Eigenmann, 1888	N	6	–
Cari	*Rhinelepis aspera* Spix & Agassiz, 1829	N	9	5
Pimelodidae				
Pirá	*Conorhynchos conirostris* Valenciennes, 1840	N	4	–
Surubim	*Pseudoplatystoma coruscans* Spix & Agassiz, 1829	N	5	–
Pseudopimelodide				
Pacamã	*Lophiosilurus alexandri* Steindachner, 1877	N	–	3
Auchenipteridae				
Caboge	*Parauchenipterus galeatus* Linnaeus, 1766	N	6	–
Heptapteridae				
Mandim	*Pimelodella* cf*. vittata* Lütken, 1874	N	4	1
Sciaenidae				
Cruvina	*Pachyurus francisci* Cuvier, 1830	N	4	–
Gymnotidae				
Sarapó	*Gymnotus* cf*. carapo* Linnaeus, 1758	N	4	–

Legend: *CA* Cabrobó, *O* Orocó, *N* native species, *E* exotic species

Considering the species richness recorded for the São Francisco River basin, which totals 244 fish species, we found that 10.3 % (*n* = 25) of these species are recognized and used by the Truká people who inhabit the villages surveyed. A total of 21 (84 %) fish species cited by interviewees as being currently fished are native, which highlights the Truká preference for these species compared to exotic species that have been introduced in the São Francisco River Basin and, according to Sato and Godinho [35], have established populations in the river. Those authors highlight the following species among the fish introduced in the area: peacock bass (*Cichla* spp.), South American silver croaker (*Plagioscion squamosissimus*), carp (*Cyprinus carpio*), African catfish (*Clarias gariepinus*), tambaqui (*Colossoma macropomum*), and tilapia (*Oreochromis* sp. and *Tilapia* sp.), three of which were mentioned by the interviewees. The production of some of these species has been encouraged among riverine inhabitants of the São Francisco River, including in the area surveyed. The São Francisco Valley Development Company (Companhia de Desenvolvimento do Vale do São Francisco-CODEVASF) provided access to technology for the farming of tambaqui (*Colossoma macropomum*) in the Cabrobó village.

The four exotic species cited by respondents were recently introduced (from the 70s onwards) [35–37], and they represent only a small fraction of part of the ichthyofauna locally used by the Truká people. Fish farming has increased in recent years in the sub-middle section of the São Francisco River, made possible by the existence of large dam reservoirs. However, aquaculture is not part of the traditional culture of indigenous fishers and involves skills and significance far from those associated with traditional fishing [38]. The nature of this situation is such that artisanal fishing in the sub-middle section of the São Francisco River, where fish has always constituted an important nutritional component for local human populations, has been declining each year.

For the interviewed fishers, food is the main objective of fishing. However, they claim that fish are currently used as a complimentary protein source, in contrast with a few decades ago, when fish accounted for a significant part of the diet in the villages surveyed. Such evidence reinforces observations reported in previous studies, which indicate that currently, the protein base of the Truká diet is composed of meat from domestic vertebrates, including cattle, goats, sheep and pigs [31]. This information suggests that there has been a change in the main protein sources of the Truká Indians. According to Aspelin and Santos [28], fishing was a very common practice among the Truká people. However, this activity has declined in importance as a result of fish shortages and the changes experienced by this ethnic group over the years through constant contact with non-Indians [39]. When there is a surplus in the fishing, the fishes are commercialized within the village among local indigenous families.

Changes in the diets of human populations resulting from factors such as urbanization and contact with nonindigenous populations have been recorded worldwide [40, 41]. Our results show that this situation has occurred among the Truká and possibly among other indigenous and nonindigenous communities who settled along the São Francisco River, which was one of the main sources of fisheries resources that supplied the fish markets of the northeast and southeast of Brazil [42].

In addition to the importance of fish as food, we recorded the use of fish in traditional Truká medicine. In Brazil, the use of animals, including fish, as medicinal resources is a fairly widespread and ancient phenomenon. Fish are among the most frequently used vertebrates for medicinal purposes in fishing and riverine communities [43, 44]. Although continental and semi-arid areas have a lower diversity of fish species in relation to the coastal areas, some of these species are used in folk medicine [45]. The use of fishes in popular medicine has been recorded in fishing communities in Brazil and many other countries in Latin America [44, 46–54]. The multiple use of fishes by humans is common [43, 55] and reinforces the importance of fish in the culture, livelihood and economic activities of fishing communities [5]. The reliance on traditional uses of animals as food and as medicine by communities around the world highlights the need for further interdisciplinary research in ethnozoology which can be used in strategies to conserve biodiversity [55] In the surveyed area, it was not registered food taboos associated to fishes mentioned by interviewees. It differs from what has been reported in previous studies [54, 56, 57] that points out food taboos in several fishing communities. For example, the consumption of some animals may be avoided because of their behavioral patterns and morphological characteristics [58], or in the belief that they are toxic [54].

The indigenous Truká fishers cited two fish species used as medicine, the trahira (*Hoplias malabaricus*) and spotted sorubim (*Pseudoplatystoma corruscans*). However, these species are mainly used for food, and at times, their by-products are used in folk medicine. The interviewees noted that trahira is used to treat earache, toothache and fatigue (asthma). The part used is the fat, which is warmed and rubbed on the affected body part. The trahira has medicinal use in several communities of the Brazilian semi-arid northeast and is one of the most important fish species in folk medicine in the region. Previous studies indicate that in addition to the whole animal, other parts of the fish are also used, including the fat, epidermal secretion, stomach, head, scales and meat. These products are prescribed to treat the following diseases: alcoholism, earache, inflammation, high cholesterol, sore throat, inflammation of the umbilical cord, bruising, ear inflammation, hearing problems, eye inflammation, urinary tract infection, deafness, asthma, muscle aches, erysipelas, wounds, bleeding, snake bite, conjunctivitis, oedema, rheumatism, glaucoma, and stroke [25, 43, 59–62]. This broad range of zootherapeutic uses and the various locations where such use has been recorded make *H. malabaricus* one of the fish species most extensively used in Brazilian folk medicine [59, 63, 64].

Regarding the spotted sorubim, in the areas surveyed, the spine is used to ‘remove anger’ (control bad temper/anxiousness) and ‘evil eye’. For this purpose, the fish spine is pounded, toasted, and then used to prepare a tea, which the patient drinks to achieve the desired effect. Costa-Neto et al. [26] also recorded the use of the spotted sorubim for the treatment of burns by artisanal fishers in the region of the middle São Francisco River; however, in this case, the part used was the fat.

### Capture techniques and general aspects of fishing among the Truká

In the villages surveyed, fishing can be practiced by men and women, although there is a predominance of the former. Children also participate in fishing when they accompany the adults during their fishing activities. The predominance of men may be explained by the fact that historically in Truká culture, as in other traditional communities, men are responsible for supporting and maintaining the family and women are responsible for caring for the home. Ethnozoological studies show that activities such as fishing and hunting, two of the main subsistence activities of indigenous communities, are practiced predominantly by men, who end up developing greater knowledge about the species exploited and their biology, as this knowledge is important in the organization of fishing and hunting activities [5, 6, 65–68].

**Fig. 2:**
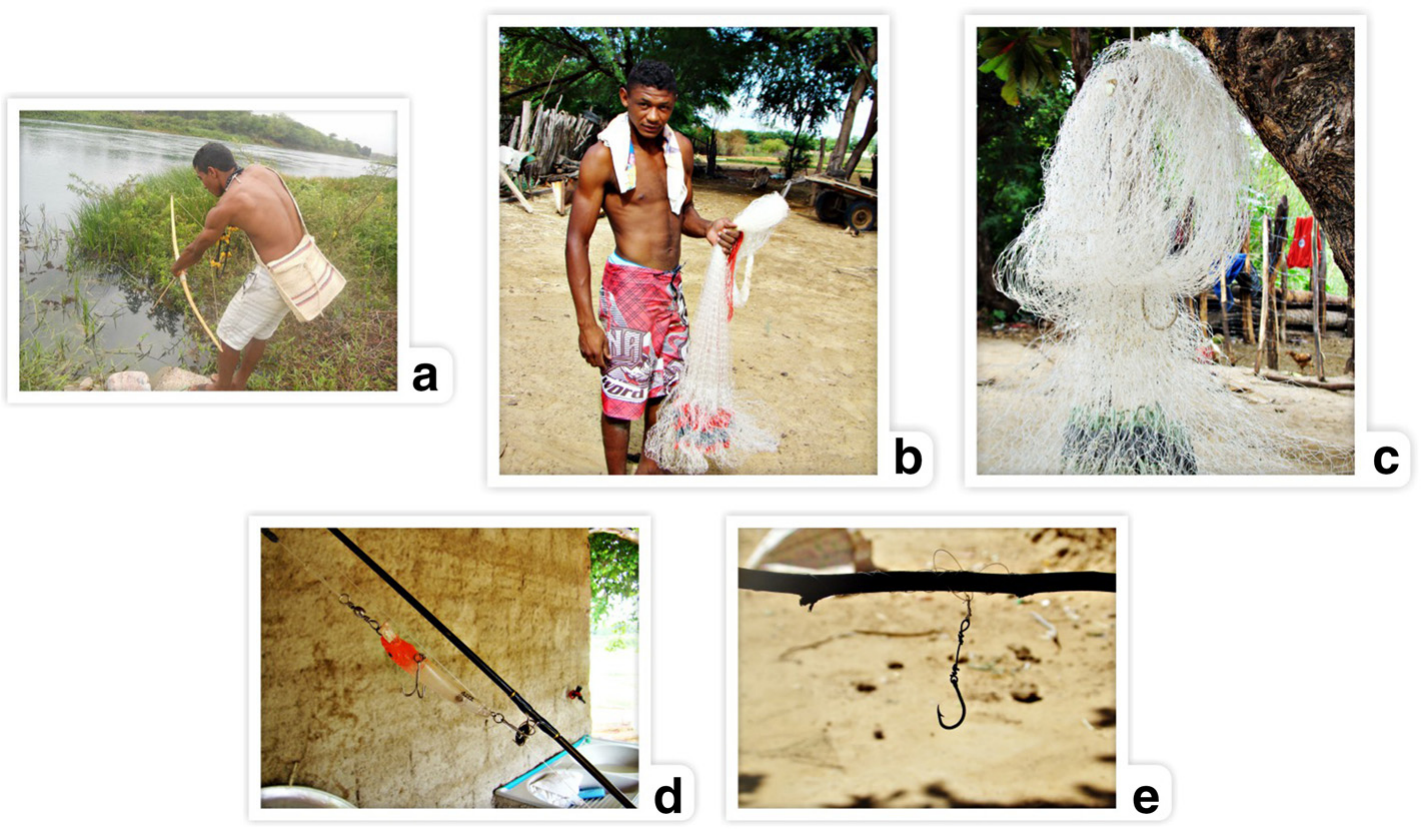
Truká fishing techniques: (**a**) bow and arrow; (**b**) cast net; (**c**) net; (**d**) hook; (**e**) hand line

To catch fish, the interviewees cited five different fishing techniques: bow and arrow, rod and hook, cast net, fishing net and hand line (Fig. 2). The first technique is one of the oldest fishing techniques practiced by indigenous communities in Brazil [69]; however, according to the interviewees, currently, it is not widely used in the surveyed area because it requires a lot of skill.

**Fig. 3:**
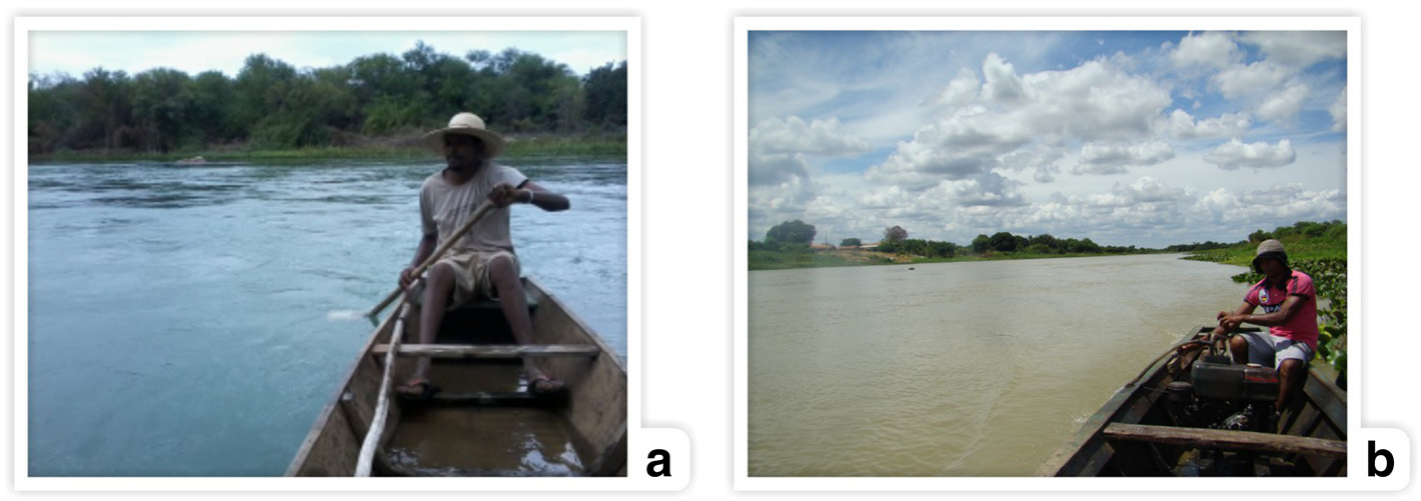
Fishing canoe. (**a**) Paddle; (**b**) Diesel engine

According to interviewees, fishing activities do not require much time in the surveyed area. This is a common characteristic in the fishing areas of the Brazilian semi-arid region, where fishing trips usually last a few hours. This situation differs from estuarine and marine fishing, which in some cases can take several hours or even days [5, 8]. In the two villages surveyed, the travel to the fishing areas in the river is performed with the aid of canoes, with paddles (Fig. 3a) or a diesel engine (Fig. 3b) used as the driving force.

### Ecological knowledge on fished species

Artisanal fishers develop an elaborate knowledge of the abiotic and biotic factors related to the fisheries resources they exploit [11, 13, 70–73]. This knowledge is important to the organization and success of fishing activities. The Truká fishers were found to have a broad knowledge of the distribution of fish species in the environment and their position in the water column, i.e., the depth the animals usually inhabit. This information is important when choosing the fishing gear to be used and for selecting the target species (Table 2). The fishing technique used is chosen while taking into consideration the target species and the depth and vertical distribution of the fish. Moreover, fishermen also recognize a diversity of fish habitats, citing waterfalls, rapids, rock caves and vegetation patches as the preferred habitats of some species (Table 3).

**Table 2:** Association between vertical habitat division, fish species and fishing artefact used by Truká fishers

Habitat	Fish species (Cabrobó)	Fish species (Orocó)	Fishing artefact
‘Water surface’ (30 cm)	–	–	–
‘Shallow’ (100 cm)	*Hoplias malabaricus*	*Hoplias malabaricus* and *Lophiosilurus alexandri*	Bow and arrow Hand line
‘Moderately deep’ (160 cm)	*Cichla ocellaris*, *Serrasalmus brandtii*, *Salminus* cf*. brasiliensis*, *Conorhynchus conirostris*, *Pachyurus Francisci*	*Serrasalmus brandtii*	Rod and hook Hand line Cast net Paddle canoe
‘Deep’ (starting at 200 cm)	*Pseudoplatystoma Coruscans*, *Pimelodella* cf*. vittata*, *Leporinus* cf *piau*, *Metynnis maculatus*, *Pterygoplichthys etentaculatus*, *Astronotus ocellatus*, *Colossoma macropomum*, *Hypostomu*s cf *margaritifer*, *Plagioscion squamosissimus*, *Brycon reinhardtii*, *Prochilodus argenteus*, *Conorhynchus conirostris*, *Pygocentrus piraya*	*Pimelodella* cf*. vittata*, *Leporinus* cf *piau*, *Metynnis maculatus*, *Cichla ocellaris*, *Colossoma macropomum*, *Hypostomu*s cf *margaritifer*, *Plagioscion squamosissimus*, *Prochilodus argenteus*, *Pygocentrus piraya*	Rod and hook Fishing net Motorized canoe
‘Mud’ (450 cm to 1000 cm)	*Hoplias malabaricus*, *Hypostomu*s *macrops*, *Pterygoplichthys etentaculatus*, *Gymnotus* cf*. carapo*, *Parauchenipterus galeatus*	*Pterygoplichthys etentaculatus*	Cast net Rod and hook Fishing net

**Table 3:** Association between river sections, fish species and fishing artefacts informed by Truká fishers

Habitat	Fish species (Cabrobó)	Fish species (Orocó)	Fishing artefact
‘Waterfalls’	*Hoplias malabaricus*, *Serrasalmus brandtii*,	*Hoplias malabaricus*	Rod and hook
	*Conorhynchus conirostris*, *Pachyurus Francisci*		
‘Rapids’	*Astronotus ocellatus*, *Colossoma macropomum*, *Hypostomus* cf *margaritifer*, *Plagioscion squamosissimus*, *Prochilodus argenteus*, *Pygocentrus piraya*, *Cichla ocellaris*	*Cichla ocellaris*, *Colossoma macropomum*, *Hypostomu*s cf *margaritifer*, *Plagioscion squamosissimus*, *Prochilodus argenteus*, *Pygocentrus piraya*	Rod and hook
‘Rock caves’	*Salminus* cf*. brasiliensis*, *Pseudoplatystoma Coruscans*, *Hypostomus* *macrops*, *Pterygoplichthys etentaculatus*, *Conorhynchus conirostris*, *Parauchenipterus galeatus*, *Gymnotus* cf*. carapo*, *Pimelodella* cf*. vittata*, *Pterygoplichthys etentaculatus*	*Serrasalmus brandtii*, *Lophiosilurus alexandri*, *Pimelodella* cf*. vittata*	Rod and hook Cast net
‘Vegetation patches’	*Leporinus* cf *piau*, *Brycon reinhardtii*, *Metynnis maculatus*	*Leporinus* cf *piau* and *Metynnis maculatus*	Cast net^a^ Fishing net Rod and hook

Legend: ^a^Cited only by the indigenous Truká fishers from Orocó

Knowledge of the distribution, habitat, and ecology of fish species is an important driver of the capture strategies to be used. A previous study conducted with fishers in the Três Marias dam and other sections of the upper-middle São Francisco also revealed that this type of knowledge is applied to the selection of fishing techniques [74]. Those authors note that the compartmentalization of the aquatic ecosystem perceived by the fishers reduce the uncertainty of fishing because fish are a mobile resource and therefore uncertain. These ethno-habitats may be understood as ecozones, which were defined by Posey [75] as ecological areas recognized in other cultural systems and may or may not reflect the scientific classification. These findings reinforce previous studies that showed the importance of knowledge of abiotic and ecological factors in the organization of fishing activities, whether in coastal or inland areas [11, 14, 70, 71, 76, 77].

### Environmental changes and their influence on fishing among the Truká

The fishers cited several problems that, according to them, have led to the decline of fishing in the villages surveyed. These problems include the growth of illegal fishing in the Truká territory by non-indigenous fishers, deforestation of the river banks, introduction of exotic species and pollution. These same factors were reported by Oliveira and Souza [36] to cause the decline of fishing stocks in the sub-middle São Francisco River. The introduction of exotic species in fresh water environments has been recognized as one of the greatest impacts to native species throughout the world [78–80]. In the case of the São Francisco River, several species have been introduced, and as indicated by the statements of the interviewees, these introductions have impacted the native species and caused changes in the traditional fishing activity of riverine populations. The species cited by the fishers included the butterfly peacock bass (*Cichla ocellaris*) and South American silver croaker (*Plagioscion squamosissimus*). These species were introduced to the Sobradinho Hydroelectric Plant Lake by the National Department of Works Against Drought (Departamento Nacional de Obras Contra as Secas-DNOCS) at the end of the 1970s [37]. In addition, many other species have been introduced species from fish farming experiments in the region, such as the oscar (*Astronotus ocellatus*) and tambaqui (*Colossoma macropomum*), generating at times negative impacts on the native fish populations [37].

The local fishers also mentioned that pollution, use of pesticides and sewage from riverine cities discharged into the river are factors that have caused the decline of native fish species. The occurrence of these problems along the São Francisco River basin has been recognized in different studies and, as highlighted by Gisler and Vasconcelos [81], has led to a reduction in fish stocks in the region.

The Truká, as well as other riverside communities along the São Francisco River, have been heavily affected by environmental degradation, which has intensified as a result of the construction of a series of dams along the course of the river and the intensification of urbanization [28, 35, 42]. Hydroelectric dams have strong negative impacts on fishing and are among the main causes of the decline of fishing in rivers and freshwater environments in many countries [82, 83]. The regulation of the hydrological regime of a river through dams is generally recognized as one of the most devastating forms of habitat degradation of inland waters [84]. Several causes may be attributed to the decline of fishing in the São Francisco River, such as pollution, improper use of soil, inadequate fishing laws, overfishing, habitat destruction and damming. All of these factors are recognized by the Truká as causing impacts and as they note, have changed the way of life not only of the Truká people but of other ethnic groups associated with the river.

## Conclusions

Our results demonstrate that fishing is still an activity that persists among the Truká, who use the considerable richness of native fish species (and some exotic ones) as a food resource. Among the species used for food, two have by-products that are used in local traditional medicine. Despite the persistence of fishing and the use of fishes by the Truká, the testimonies of the interviewees indicated that this activity has less importance to their subsistence than in the past and that this shift has taken place due to a series of factors that have impacted the São Francisco River and caused the decline of fish stocks in the communities surveyed. This situation is an example of how environmental degradation has affected the subsistence culture of indigenous communities.

Although fishing is declining in importance among the Truká, we found that the individuals who are still practicing this activity have a broad knowledge about the habitat and ecology of the target species and apply that knowledge to fishing methods. The Truká also provided information on the impacts related to population declines in the fish fauna of the São Francisco River. Knowledge about the ecology of the species and the environmental impacts that have affected them can support basic research on local fish populations and research investigating the environmental impacts, resource management and sustainable exploitation of fisheries resources.
