# Erratum to: Ethnoichthyology of the indigenous Truká people, Northeast Brazil

**DOI:** 10.1186/s13002-016-0078-y

**Published:** 2016-01-14

**Authors:** Carlos Alberto Batista Santos, Rômulo Romeu Nóbrega Alves

**Affiliations:** Programa de Pós Graduação em Etnobiologia e Conservação da Natureza, Departamento de Ciências Biológicas, Universidade Federal Rural de Pernambuco, Rua Dom Manoel de Medeiros, s/n, Dois Irmãos, 52171-900 Recife, PE Brazil; Departamento de Tecnologia e Ciências Sociais, Universidade do Estado da Bahia, Avenida Edgard Chastinet, s/n, São Geraldo, 48905-680 Juazeiro, BA Brazil; Departamento de Biologia, Universidade Estadual da Paraíba, Av. das Baraúnas, 351/Campus Universitário, Bodocongó, 58109-753 Campina Grande, Paraíba Brazil

## Erratum

The original version of this article [1] unfortunately contained a mistake. In the author list, the surname of author Rômulo Romeu Nóbrega Alves was incorrectly spelled. The original article has now been updated with the correct spelling.
